# Demographic and disease‐related factors impact on cerebrospinal fluid neurofilament light chain levels in multiple sclerosis

**DOI:** 10.1002/brb3.2873

**Published:** 2022-12-27

**Authors:** Kamila Zondra Revendova, Chiara Starvaggi Cucuzza, Ali Manouchehrinia, Mohsen Khademi, Michal Bar, David Leppert, Elisabeth Sandberg, Russell Ouellette, Tobias Granberg, Fredrik Piehl

**Affiliations:** ^1^ Centre for Molecular Medicine, Department of Clinical Neuroscience Karolinska Institutet Stockholm Sweden; ^2^ Centre for Neurology, Academic Specialist Center Karolinska University Hospital Stockholm Sweden; ^3^ Department of Clinical Neurosciences Faculty of Medicine University of Ostrava Ostrava Czech Republic; ^4^ Neurologic Clinic and Policlinic, MS Center and Research Center for Clinical Neuroimmunology and Neuroscience Basel (RC2NB) Basel Switzerland; ^5^ Department of Neuroradiology Karolinska University Hospital Stockholm Sweden

**Keywords:** biomarker, multiple sclerosis, neurofilament light chain, progressive multiple sclerosis, relapsing‐remitting multiple sclerosis, disease‐modifying therapies, brain atrophy

## Abstract

**Background:**

Neurofilament light (NfL) levels reflect inflammatory disease activity in multiple sclerosis (MS), but it is less clear if NfL also can serve as a biomarker for MS progression in treated patients without relapses and focal lesion accrual. In addition, it has not been well established if clinically effective treatment re‐establishes an age and sex pattern for cerebrospinal fluid NfL (cNfL) as seen in controls, and to what degree levels are affected by disability level and magnetic resonance imaging (MRI) atrophy metrics.

**Methods:**

We included subjects for whom cNfL levels had been determined as per clinical routine or in clinical research, classified as healthy controls (HCs, *n* = 89), MS‐free disease controls (DCs, *n* = 251), untreated MS patients (uMS; *n* = 296), relapse‐free treated MS patients (tMS; *n* = 78), and ProTEct‐MS clinical trial participants (pMS; *n* = 41).

**Results:**

Using linear regression, we found a positive association between cNfL and age, as well as lower concentrations among women, in all groups, except for uMS patients. In contrast, disability level in the entire MS cohort, or T1 and T2 lesion volumes, brain parenchymal fraction, thalamic fraction, and cortical thickness in the pMS trial cohort, did not correlate with cNfL concentrations. Furthermore, the cNfL levels in tMS and pMS groups did not differ.

**Conclusions:**

In participants with MS lacking signs of inflammatory disease activity, disease modulatory therapy reinstates an age and sex cNfL pattern similar to that of control subjects. No significant association was found between cNfL levels and clinical worsening, disability level, or MRI metrics.

## Graphical abstract

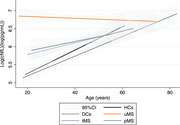

## 1 INTRODUCTION

Multiple sclerosis (MS) is a chronic inflammatory demyelinating and neurodegenerative disease of the central nervous system in which extent of neuroaxonal damage represents a major determinant of permanent disability (Filippi et al., 2018). One of the main neuroaxonal cytoskeletal proteins is the neurofilament light chain (NfL), which upon axonal damage is released into the cerebrospinal fluid (CSF) and can be detected at much lower concentrations also in peripheral blood (Bittner et al., 2021). Consequently, increased levels of CSF NfL (cNfL) have been described in a wide spectrum of degenerative neurological conditions, including MS (Bridel et al., 2019; Martin et al., 2019; Momtazmanesh et al., 2021). An increasing body of evidence suggests that NfL levels is a proxy for ongoing inflammatory disease activity, as reflected by relapses and accrual of focal lesions detected by magnetic resonance imaging (MRI) (Khalil et al., 2018). However, the capacity of NfL to capture the progressive aspect of MS is less clear (Kapoor et al., 2020). It is also striking that while a correlation between cNfL levels and age is readily evident in healthy controls (HCs), this pattern was not observed in a large meta‐analysis including a large group of treated and untreated MS patients (Bridel et al., 2019). Furthermore, there was a similar sex difference, with lower cNfL levels in women, in both HCs and MS (Bridel et al., 2019), not observed in serum (Benkert et al., 2022). Due to being less invasive and therefore better adapted for regular monitoring purposes, major efforts have been invested in developing techniques for detecting NfL in blood (Khalil et al., 2018). However, unlike cNfL levels, blood NfL concentrations can be influenced by other factors such as body mass index, blood volume, kidney function, cardiovascular diseases, smoking, or drug‐induced neurotoxicity (Akamine et al., 2020; Alagaratnam et al., 2021; Barro et al., 2020; Ladang et al., 2022; Manouchehrinia et al., 2020). Nevertheless, an interesting observation is that highly effective disease‐modifying therapies (DMTs), in particular B‐cell‐depleting therapies, have been shown to normalize blood NfL levels in both relapsing‐remitting and progressive MS diseases (Bar‐Or et al., 2019). In fact, in a recent large‐scale study, highly effective monoclonal DMTs tended to lower blood NfL levels beyond those seen in a normative reference population (Benkert et al., 2022). The reason for this is unclear, but it may be speculated that the extent of accumulated neuroaxonal loss, in particular of long tract connections, may result in lower steady state NfL release once inflammatory disease activity has been curbed.

The main objective was to determine whether treated MS patients lacking signs of inflammatory disease activity showed a similar age and sex pattern for cNfL as seen in controls. Second, the relationships between cNfL and disability, progressive phenotype of MS, MRI metrics for lesion burden, and brain atrophy were evaluated.

## 2 METHODS

### 2.1 Study design and participants

A single‐center retrospective multi‐cohort study was conducted at the Centre for Neurology, Academic Specialist Center, Karolinska University Hospital, and at the Centre for Molecular Medicine, Karolinska Institutet, Stockholm, Sweden. We used three sources to obtain participant's data: the Swedish MS Registry (Alping et al., 2019), medical records, and archived data in the CSF biobank at the Department of Neurology, Karolinska University Hospital.

The Swedish MS Registry was used to identify MS patients attending the Centre for Neurology, Academic Specialist Center with available results of cNfL concentrations sampled from January 1, 2012 to December 31, 2021. Patients were grouped according to ongoing treatment at the time of sampling in untreated MS (uMS) and treated MS (tMS) cases. Most of the patients in the cohort with ongoing DMT had volunteered to perform a lumbar puncture for research purposes or did so as a routine check. We excluded tMS patients with DMTs less than 6 months before the lumbar puncture, those with a recorded relapse within 3 months of CSF sampling, and when CSF collection was prompted due to comorbidity. The diagnosis of MS was established according to the 2017 McDonald criteria (Thompson et al., 2018).

As a separate cohort, we included baseline clinical, CSF, and MRI data for participants in the ProTEct‐MS study (pMS), a single‐center phase II randomized controlled trial (clinicaltrial.gov identifier NCT04480307) investigating the add‐on effect of temelimab in relapsing‐remitting MS (RRMS) patients treated with rituximab for at least 1 year. Patients were included in this trial if presenting with clinical worsening in (i) one or more neurological domains as assessed by the Expanded Disability Status Scale (EDSS) score (Kurtzke, 1983), (ii) ambulatory function as assessed by 6 min or timed 25 feet walk test, (iii) cognitive functioning as assessed by the symbol digit modalities test, or (iv) increased need of walking aids or pharmacological/procedures for bowel and bladder functions over the last year. Further inclusion criteria were as follows: age 18–55 years (inclusive), an EDSS of 2.5–5.5 (inclusive), >9 T2 cerebral lesions, a B‐cell count below the lower limit of detection, and a stable clinical status for 30 days prior to screening. Primary progressive MS (PPMS) was an exclusion criterion. For full inclusion and exclusion criteria, see Table S1. Subjects were enrolled between June 17, 2020 and February 4, 2021.

Data on MS‐free control subjects having undergone CSF sampling from January 1, 2002 to February 30, 2022 were extracted from the Karolinska CSF biobank. Patients lacking a specific diagnosis were excluded. Study controls were categorized according to the BioMS‐eu consortium (Teunissen et al., 2013) to HCs, symptomatic controls and non‐inflammatory disease controls, but for the purpose of this study, we considered symptomatic and non‐inflammatory disease controls jointly as disease controls (DCs). For details about study controls, see Table S2.

### 2.2 Demographic and clinical data

We collected demographic and clinical data about age, sex, DMTs history, MS disease history, clinical relapses, and the EDSS and functional systems (FS) scores from the Swedish MS Registry. EDSS in the uMS and tMS cohorts had been performed by the attending neurologist and did not always include FS scores. In the trial cohort, EDSS had been performed by certified EDSS raters and had been obtained 1–7 days prior to lumbar puncture. Disease duration was defined as time from the date of disease onset to the date of CSF sampling.

Ongoing and previous DMTs were categorized into moderately effective DMTs (all interferons, dimethyl fumarate, glatiramer acetate, and teriflunomide), highly efficacious DMTs (HET: alemtuzumab, cladribine, fingolimod, natalizumab, ofatumumab, ocrelizumab, and rituximab), others (registered as “study drugs” or “others” in the Swedish MS Registry), and autologous hematopoietic stem cell transplantation (aHSCT).

### 2.3 MRI scans and analysis

Brain MRI scans for the clinical trial cohort were performed within 1–7 days prior to CSF sampling on a Siemens Prisma^Fit^ 3.0 Tesla MRI scanner (Siemens Healthineers, Erlangen, Germany) using a clinical 64‐channel head coil. The study protocol included 3D (1 mm isotropic) acquisitions of T1‐weighted imaging and T2‐weighted Fluid‐Attenuated Inversion Recovery (FLAIR) imaging. The 3D T1‐weighted acquisition was processed using FreeSurfer (v. 7.0, http://freesurfer.net) to isolate the T1 lesion volume, brain parenchymal fraction (BPF), thalamic volume (normalized by the intracranial volume to the thalamic fraction), and cortical thickness (FreeSurfer, 2012). The T2‐weighted FLAIR lesion volume was extracted using Lesion Segmentation Toolbox (v2.0.15, Technische Universität München, Munich, Germany, applied‐statistics.de/lst) (Schmidt et al., 2012).

### 2.4 Samples and analytical methods

CSF samples were collected into polypropylene tubes, centrifuged at 350g for 10 min at room temperature immediately after sampling and the supernatant was stored at −80°C until analysis. Concentrations of cNfL were determined with commercially available enzyme‐linked immunosorbent assay (ELISA) kits (Uman Diagnostics, Umeå, Sweden) either at the Centre for Molecular Medicine or at the Department of Clinical Immunology, Karolinska University Hospital, according to the manufacturers’ instructions. Measurements were performed in duplicate using 50 μl of undiluted cell‐free CSF per duplicate. The detection limit was 33 pg/ml. Intra‐assay coefficients of variation were below 5%, and inter‐assay coefficients of variation were below 10%.

### 2.5 Statistical analysis

Descriptive statistics were conducted to describe baseline characteristics. Quantitative data are reported as medians and interquartile ranges (IQR), while qualitative data are reported as counts and percentages. Kruskal–Wallis equality‐of‐populations rank test was used to compare cNfL levels across groups, and Dunn's pairwise test with Bonferroni adjustment was performed for comparisons between each independent group. Simple and multiple linear regressions were used to determine the association between cNfL (dependent variable) and demographic, clinical, and radiological variables (independent variables), where cNfL values were logarithmically transformed because of non‐normal distribution and to reduce the impact of outliers. The significance level was set at 5%. All analyses were conducted with Stata Statistical Software v17 (StataCorp LLC, College Station, TX).

### 2.6 Ethical approval

Written informed consent was obtained from all study participants and the Regional Ethics Review Board in Stockholm and Swedish Ethical Review Authority approved the procedures reported in this work (for MS patients, HCs and DCs, No. 2009/2017‐31/2, last amendment 2021–02060; for the trial cohort, 2020‐00557).

## 3 RESULTS

### 3.1 Demographic and cNfL levels

**FIGURE 1:**
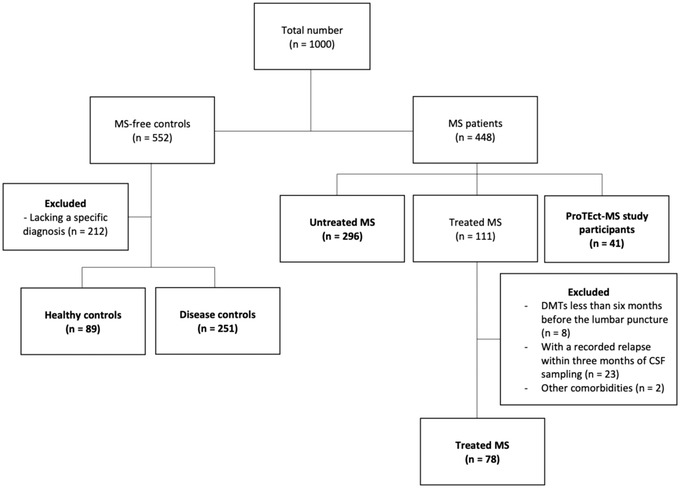
Study flowchart. Abbreviations: CSF, cerebrospinal fluid; DMTs, disease modifying therapies; MS, multiple sclerosis; *n*, number

Data from a total of 755 participants were included in the analyzed data set, which comprised 89 HCs, 251 DCs, and 415 MS patients, of whom 296 were uMS, 78 tMS, and 41 were pMS clinical trial participants. Baseline characteristics are detailed in Table 1, and the study inclusion flow chart is shown in Figure 1. Proportion of females and age at sampling were similar across disease controls and the larger MS cohort, while HCs and pMS differed consistently. Specifically, only around 50% of subjects were female in the HCs and pMS groups (compared to 64%–66% in DCs, uMS, and tMS) and median ages of HCs and pMS were lower and higher, respectively, than those of DCs and the larger MS cohort. Untreated MS and tMS patients were similar regarding disease characteristics, such as clinical phenotype and EDSS disability level, while disease duration as expected was shorter in uMS. Participants of the clinical trial were more likely to have SPMS and had longer disease duration and higher disability level.

**TABLE 1:** Demographic, clinical, and magnetic resonance imaging (MRI) characteristics of the study cohorts

Cohort	HCs	DCs	uMS	tMS	pMS
N	89	251	296	78	41
Sex (*n*, % female)	44 (49.4)	166 (66.1)	191 (64.5)	50 (64.1)	22 (53.7)
Age at sampling (median years, IQR)	27 (23–32)	39 (30–52)	34 (27–45)	34 (29–44)	47 (42–51)
cNfL level (median pg/ml, IQR)	260 (190–330)	300 (177–490)	780 (495–1365)	420 (310–570)	510 (410–620)
Disease duration at sampling (median years, IQR)			1.00 (0.17–5.09)	4.9 (2.6–9.6)	13.5 (7.6–19.6)
MS phenotype (*n*, %)					
RRMS			260 (87.8)	72 (92.3)	25 (61.0)
PPMS			14 (4.7)	0 (0.00)	0 (0.0)
SPMS			22 (7.4)	6 (7.69)	16 (39.0)
Duration of DMTs (median months, IQR)				18 (8–34)	51 (25–72)
DMTs category (*n*, %)					
Moderately effective DMTs				16 (20.5)	
Highly effective DMTs				56 (71.8)	41 (100)
aHSCT				6 (7.7)	
Previous DMTs (*n*, %)					
Moderately effective DMTs			23 (7.8)	15 (19.2)	8 (19.5)
Highly effective DMTs			2 (0.7)	14 (18.0)	11 (26.8)
Others			3 (1.01)	1 (1.3)	7 (17.1)
EDSS at sampling (median, IQR)			2.0 (1.0–2.5)*	2.0 (1.0–3.0)**	4.0 (3.0–4.0)
Ambulation score (median, IQR)					1 (1–1)
Bowel and bladder FS (median, IQR)					1 (0–2)
Pyramidal FS (median, IQR)					3 (2–3)
Sensory FS (median, IQR)					2 (2–3)
T1 lesion volumes (median, IQR)^†^					2.37 (1.23–4.13)
T2 lesion volumes (median, IQR) ^†^					7.88 (3.06–16.91)
Brain parenchymal fraction (median, IQR) ^†^					0.76 (0.74–0.78)
Thalamic fraction (median, IQR) ^†^					0.01 (0.01–0.01)
Cortical thickness (median, IQR) ^†^					2.75 (2.69–2.81)

Abbreviations: aHSCT, autologous hematopoietic stem cell transplantation; cNfL, cerebrospinal fluid neurofilament light chain; DCs, disease controls; DMTs, disease modifying therapies; EDSS, Expanded Disability Status Scale; FS, functional system score; HCs, healthy controls; N, number; pMS, ProTEct‐MS study participants; PPMS, primary progressive multiple sclerosis; RRMS, relapsing‐remitting multiple sclerosis; SPMS, secondary progressive multiple sclerosis; tMS, treated MS patients; uMS, untreated MS patients.

^†^ Only 40 patients.

* Only 283 patients.

** Only 71 patients.

The median cNfL level was 260 pg/ml (IQR 190–330) in HCs, 300 pg/ml (IQR 177–490) in DCs, and 630 pg/ml (IQR 430–1040) in MS patients. These diagnostic groups were significantly different from each other according to Kruskal–Wallis rank test and pairwise post hoc Dunn's test (HCs vs. DCs *p* = .006, HCs vs. MS *p* < .001, and DCs vs. MS patients *p* < .001). In uMS patients, the median cNfL concentration was 780 pg/ml (IQR 495–1365 pg/ml), in tMS 420 pg/ml (IQR 310–570 pg/ml), and in pMS 510 pg/ml (IQR 410–620 pg/ml). Significant differences in cNfL levels between uMS and tMS and between uMS and pMS (*p* < .001), but not between tMS and pMS (*p* = .26), were identified by Dunn's pairwise test. In contrast, cNfL concentrations did not differ between MS phenotypes (Kruskal–Wallis rank test *p* = .54). In addition, variability in cNfL levels tended to be greater in disease controls and uMS compared with HC, tMS, and pMS across age strata (data not shown).

### 3.2 Association between cNfL and age and sex across all groups

**FIGURE 2:**
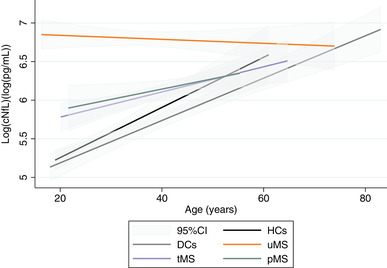
Cerebrospinal neurofilament light chain between diagnostic groups according to age. The age predicted cNfL levels in healthy controls (*p* < .001), disease controls (*p* < .001), treated MS (*p* = .001), and ProTEct‐MS (*p* = .028), but not in untreated MS (*p* = .536). Abbreviations: 95% CI, 95% confidence interval; cNfL, cerebrospinal fluid neurofilament light chain; DCs, disease controls; HCs, healthy controls; uMS, untreated MS; pMS, the ProTEct‐MS study participants; tMS, treated MS

Simple linear regression showed a positive association between age and cNfL levels in HCs (*ß* = 0.03, 95% confidence interval [CI] 0.02–0.04), DCs (*ß* = 0.03, 95% CI 0.02–0.03), tMS (*ß* = 0.02, 95% CI 0.01–0.03), and pMS (*ß* = 0.01, 95 % CI 0.002–0.03). In contrast, in uMS patients, there was no association with age (*ß* = −0.003, 95% CI −0.01 to 0.01) (Figure 2). Adjustment for sex and disease characteristics (for MS groups) did not substantially change the results (Table 2). When evaluating the impact of sex, we found lower values adjusted for age in women across all cohorts (*ß* = −0.20, 95% CI −0.37 to −0.03; *ß* = −0.51, 95% CI −0.71 to −0.32; *ß* = −0.22, 95% CI −0.42 to −0.02; *ß* = −0.20, 95% CI −0.39 to −0.02; in HCs, DCs, tMS, and pMS, respectively) except for uMS (*ß* = −0.18, 95% CI −0.38 to 0.03).

**TABLE 2:** Associations between cerebrospinal fluid neurofilament light chain and age, and sex across diagnostic groups. Simple linear regression model included age, multiple regression model 1 included age and sex, and model 2 included age, disease duration, Expanded Disability Status Scale (EDSS), and sex

Group	N	Variable	Simple model			Multiple model 1			Multiple model 2		
			*ß* (95% CI)	*R* ^2^	*p*‐Value	*ß* (95% CI)	*R* ^2^	*p*‐Value	*ß* (95% CI)	*R* ^2^	*p*‐Value
HCs	89	Age	0.03 (0.02, 0.04)	0.27	<.001	0.03 (0.02, 0.04)	0.31	<.001	NA	NA	NA
	89	Female				‐0.20 (−0.37, −0.03)	0.31	.019	NA	NA	NA
DCs	251	Age	0.03 (0.02, 0.03)	0.20	<.001	0.03 (0.02, 0.03)	0.28	<.001	NA	NA	NA
	251	Female				–0.51 (−0.71, −0.32)	0.28	<.001	NA	NA	NA
uMS	296	Age	–0.003 (−0.01, 0.01)	0.00	.536	–0.003 (−0.01, 0.01)	0.01	.475	0.01 (−0.01, 0.02)	0.06	.316
	296	Female				–0.18 (−0.38, 0.03)	0.01	.096	–0.16 (−0.01, 0.16)	0.06	.136
tMS	78	Age	0.02 (0.01, 0.03)	0.14	.001	0.02 (0.01, 0.02)	0.19	.001	0.02 (0.01, 0.03)	0.34	<.001
	78	Female				–0.22 (−0.42, −0.02)	0.19	.028	–0.24 (−0.44, −0.04)	0.34	.017
pMS	41	Age	0.01 (0.002, 0.03)	0.12	.028	0.01 (0.003, 0.03)	0.22	.015	0.01 (0.002, 0.03)	0.23	.025
	41	Female				–0.20 (−0.39, −0.02)	0.22	.035	–0.20 (−0.40, −0.01)	0.23	.041

Abbreviations: 95% CI, 95% confidence interval; DCs, disease controls; HCs, healthy controls; N, number; pMS, ProTEct‐MS study participants; tMS, treated MS patients; uMS, untreated MS patients.

### 3.3 Association between cNfL and disability level and disease duration in MS patients

**FIGURE 3:**
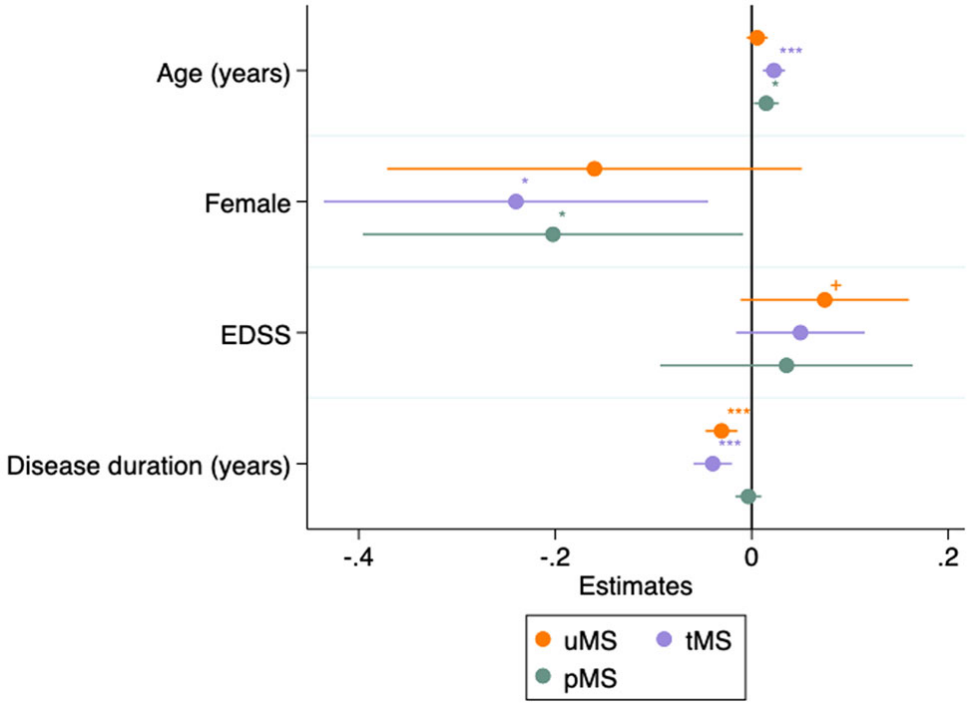
Coefficient plot with 95% confidence interval from the multiple linear regression, where cNfL is an outcome, and age, women as reference group, EDSS and disease duration in years are predictors. Legend: In treated MS and ProTEct‐MS, there was a positive association between age and cNfL levels and lower cNfL levels in women. In untreated MS, there was no association with age and sex. The EDSS score did not affect the cNfL levels in all MS groups. The disease duration was negatively associated with cNfL concentrations in treated MS and untreated MS, but not in ProTEct‐MS study group. +*p*‐value < .1, *p‐value < .05, **p‐value < .01, ***p‐value < .001. Abbreviations: 95% CI, 95% confidence interval; pMS, the ProTEct‐MS study participants; tMS, treated MS; uMS, untreated MS

In simple and multiple regression models (adjusted for age, disease duration and sex), disability level as determined by the EDSS score did not associate with cNfL levels in any of the MS cohorts (Figure 3 and Table 3). Similarly, individual functional systems, such as pyramidal, sensory, sphincter, or ambulatory scores, did not influence cNfL levels in the pMS group (Table 3). In contrast, longer disease duration was associated with lower cNfL levels in both uMS (*ß* = −0.03, 95% CI −0.05 to −0.01) and tMS (*ß* = −0.04, 95% CI −0.06 to −0.02), but not in pMS (*ß* = −0.003, 95% CI −0.02 to 0.01) (Figure 3).

**TABLE 3:** Associations between cerebrospinal fluid neurofilament light chain and disease duration, and disability across multiple sclerosis (MS) groups

Diagnostic group	N	Variable	Simple model			Multiple model^a^		
			*ß* (95% CI)	*R* ^2^	*p*‐Value	*ß* (95% CI)	*R* ^2^	*p*‐Value
uMS	293	Disease duration				−0.03 (−0.05, −0.01)	0.06	<.001
	293	EDSS	−0.004 (−0.07, 0.06)	0.00	.89	0.07 (−0.01, 0.16)	0.06	.09
tMS	71	Disease duration				−0.04 (−0.05, −0.02)	0.34	<.001
	71	EDSS	0.05 (−0.02, 0.11)	0.03	.13	0.05 (−0.06, −0.02)	0.34	.14
pMS	41	Disease duration				−0.003 (−0.02, 0.01)	0.23	.60
	41	EDSS	0.07 (−0.05, 0.19)	0.04	.24	0.04 (−0.09, 0.16)	0.23	.58
	41	Ambulation score	0.06 (−0.04, 0.15)	0.04	.23	0.03 (−0.07, 0.12)	0.22	.58
	41	Bowel and bladder FS	−0.06 (−0.15, 0.03)	0.05	.16	−0.04 (−0.13, 0.39)	0.24	.29
	41	Pyramidal FS	0.04 (−0.10, 0.19)	0.01	.57	0.03 (−0.10, 0.17)	0.22	.62
	41	Sensory FS	−0.02 (−0.14, 0.09)	0.00	.70	−0.06 (−0.17, 0.04)	0.25	.23

Abbreviations: 95% CI, 95% confidence interval; N, number; pMS, the ProTEct‐MS study participants; tMS, treated MS patients; uMS, untreated MS patients.

^a^ Multiple regression model with EDSS, and disease duration included age, and sex. Other models of the ProTEct‐MS study participants adjusted only for age and sex.

### 3.4 Association between cNfL and clinical worsening

To explore the impact on cNfL levels of clinical worsening in the ProTEct‐MS group, we considered MS group as adjunctive independent variable in our multiple regression model. Using tMS patients as reference group and adjusting for age, sex, disease duration, and EDSS, we found a significant difference between tMS and uMS (*ß* = 0.71, 95% CI 0.52–0.92), but not between tMS and pMS (*ß* = 0.18, 95% CI −0.12 to 0.49).

### 3.5 Association between cNfL and MRI metrics in ProTEct‐MS participants

**FIGURE 4:**
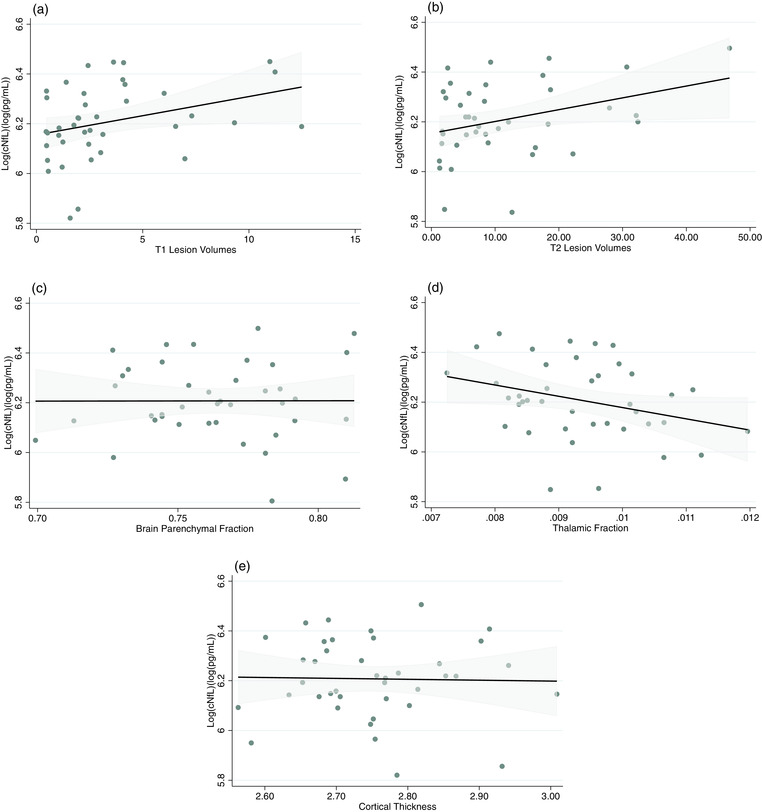
Regression plot for predicted cerebrospinal fluid neurofilament light chain values and (a) T1 and (b) T2 lesion volumes, (c) brain parenchymal fraction, (d) thalamic fraction, and (e) cortical thickness. Age and sex were included as covariates in the regression model. The shaded blue represents the 95% confidence interval.

In the clinical trial cohort, there were no statistically significant associations between cNfL levels and MRI measures, which included T2 and T1 lesion volumes, BPF, thalamic fraction, and cortical thickness (Table 4 and Figure 4).

**TABLE 4:** Associations between cerebrospinal fluid neurofilament light chain and brain volumetric measurements in the ProTEct‐MS study participants

Variable		N		Simple		Multiple*	
		*ß* (95% CI)	*R* ^2^	*p*‐Value	*ß* (95% CI)	*R* ^2^	*p*‐Value
T1 lesion volumes	40	0.02 (−0.02, 0.05)	0.02	.35	0.003 (−0.07, 0.12)	0.22	.58
T2 lesion volumes	40	0.005 (−0.05, 0.01)	0.03	.33	0.002 (−0.01, 0.01)	0.22	.82
Brain parenchymal fraction	40	0.01 (−3.78, 3.81)	0.00	.99	1.78 (−1.86, 5.42)	0.24	.33
Thalamic fraction	40	−45.3 (−144.2, 53.5)	0.02	.36	−39.13 (−129.9, 51.6)	0.24	.39
Cortical thickness	40	−0.04 (−1.07, 0.10)	0.00	.94	0.53 (−0.47, 1.52)	0.24	.29

Abbreviations: 95% CI: 95% confidence interval; N: number.

* Adjusted for age and sex.

## 4 DISCUSSION

In this large, observational, retrospective study, we observed that MS patients with ongoing DMTs display a similar age and sex pattern as healthy individuals and disease controls. In contrast, we did not demonstrate any association between cNfL concentrations and disability level or volumetric brain MRI metrics in MS participants.

The finding of a reinstated age effect on cNfL levels in treated MS patients contrasts with the largest meta‐analysis of cNfL performed to date. Bridel et al. (2019) found only a weak positive trend between cNfL levels and age in clinically isolated syndrome (CIS) and secondary progressive MS (SPMS), but not for treated or untreated RRMS and PPMS. We deem it likely that this discrepancy between their results and our findings might be explained by the composition of the studied cohorts; with enrichment of individuals with remaining inflammatory disease activity in the study by Bridel et al. In comparison, treated MS patients with clinical signs of inflammatory disease activity were excluded from analysis in our study. Additionally, only a small portion was treated with moderately effective DMTs in our cohort.

A recent large study demonstrated that highly effective DMTs lower blood NfL at the same level, or even below levels seen in control subjects (Benkert et al., 2022). It may be speculated if this phenomenon might be explained by lower steady state NfL levels due to fewer remaining neuroaxonal connections. To explore this hypothesis, we studied the correlation between cNfL levels and clinical or neuroradiological measures of accumulated damage, such as EDSS, MRI lesion volumes, and brain volume fractions. Contrary to our expectations, we were unable to detect a clear relationship between cNfL and disability status or brain atrophy, though this is also in agreement with a recent study by Kosa et al., where, however, treatment status or multiple MRI metrics were not considered (Kosa et al., 2022). It is still important to acknowledge that we cannot rule out that these factors may work in opposite directions, that is, where a lower steady state level of NfL release with increasing brain atrophy cancels out the effect of a modestly increased rate of neuroaxonal degeneration with more advanced disease. In line with the original hypothesis, although other causes must also be considered, lower levels among women may be explained by a relation between the volume of the central nervous system and steady state cNfL levels. Interestingly, we observed a negative association with disease duration in both treated and untreated MS patients, which concurs with existing data demonstrating that lowered inflammatory disease activity over time from a clinical (Kalincik et al., 2013), pathological (Frischer et al., 2015), and therapeutic response perspective (Signori et al., 2015).

A number of studies have explored the association between cNfL levels and different volumetric MRI metrics. For example, Schneider et al. (2021) recently reported that cNfL levels negatively correlated with brain gray matter (but not spinal cord) volume. Two studies, including CIS patients, reported conflicting results. Tortorella et al. (2018) found an inverse correlation between cNfL levels and total and peripheral gray matter volumes. In contrast, Khalil et al. (2013) did not find any correlation between cNfL and baseline normalized volumes of the whole brain, gray matter, white matter, cortical gray matter, and ventricular CSF. A recent study by Bhan et al., including RRMS and SPMS, found a significant association of cNfL with baseline MRI volumes of the thalamus and nucleus accumbens, and total volume of T1 and T2 lesions. However, only 17% patients were treated with DMTs at the time of lumbar puncture (Bhan et al., 2021).

In this study, we did not detect any clear association between cNfL concentrations and brain volumetric measures in patients enrolled in the ProTEct‐MS trial. It should be noted that an important difference between most of the previous studies and our cohort is that all included trial subjects at the time of sampling were on highly effective DMTs and had not displayed recent signs of inflammatory disease activity as reflected by relapses or focal MRI activity. Nevertheless, all patients had previous evidence of disease progression during the last year prior to enrollment. Taken together with previous observations obtained from cohorts with none or less effective DMTs, it seems likely that this reported associations between cNfL and volumetric MRI measures may be explained by lingering inflammatory disease activity rather than the progressive phenotype per se. It therefore seems that NfL alone is not well suited to record treatment effects in progressive MS, while still being useful to exclude residual inflammatory disease activity (Kapoor et al., 2020). Apart from NfL, there are other candidate biomarkers in MS that may be more useful as biomarkers of progression. In particular, this regards to the glial fibrillary acidic protein (GFAP), which is a promising biomarker of disease progression, with the highest concentration in patients with marked neurological disability (Axelsson et al., 2011), and chitinase 3‐like 2, which is associated with long‐term disability progression in progressive MS patients (Comabella et al., 2021).

This study has certain limitations. The larger cohort was collected in real‐world conditions with its inherent limitations on completeness and quality of collected data. The fact that MRI examinations were not coordinated with CSF samplings, except in trial subjects, meant that we could not exclude the presence of inflammatory neuroradiological disease activity at the time of sampling. As detailed MRI metrics were available only for a smaller subgroup of subjects, extrapolations of the relation between cNfL levels and brain atrophy to the general MS population should be done with caution. However, a recent study conducted in a larger group of MS patients indeed suggest that the relation between total brain parenchymal fraction and cNfL is weak or non‐existing (Kosa et al., 2022). Furthermore, the majority of cNfL concentrations had been determined in clinical routine and not in a single run. However, the clinical immunology laboratory is certified according to international standards required for reporting data for clinical use.

## 5 CONCLUSIONS

MS patients on DMT without signs of neuroinflammatory activity, as reflected by relapses or new focal MRI lesions, display similar cNfL age and sex patterns as HCs and DCs. In contrast, progressive phenotype, disability level, or brain atrophy MRI metrics were not associated with cNfL. Therefore, while cNfL remains a valid biomarker for reflecting the inflammatory aspects of MS, additional biomarkers that can better capture neurodegenerative disease mechanisms must be investigated.

## AUTHOR CONTRIBUTIONS

*Conceptualization, formal analysis, funding acquisition, investigation, methodology, project administration, visualization, and writing—original draft*: Kamila Revendova. *Conceptualization, formal analysis, methodology, visualization, and writing—original draft*: Chiara Starvaggi Cucuzza. *Formal analysis, methodology, visualization, and writing—original draft*: Ali Manouchehrinia. *Investigation and writing—original draft: Mohsen Khademi. Supervision and writing—review and Editing*: Michal Bar. *Funding acquisition, supervision, and writing—review and editing*: David Leppert. *Investigation and writing—review and editing*: Elisabeth Sandberg. *Investigation, funding acquisition and writing—original draft*: Russell Ouellette. *Investigation, funding acquisition and writing—original draft*: Tobias Granberg. Conceptualization, *Funding acquisition, investigation, methodology, project administration, supervision, and writing—original draft*: Fredrik Piehl.

## CONFLICT OF INTEREST

David Leppert is Chief Medical Officer of GeNeuro. Tobias Granberg is the recipient of a Multiple Sclerosis Innovation Award from Merck. Fredrik Piehl has received research grants from Janssen, Merck KGaA and UCB, and fees for serving on DMC in clinical trials with Chugai, Lundbeck and Roche, and for preparation of expert witness statement for Novartis. Kamila Zondra Revendova, Chiara Starvaggi Cucuzza, Ali Manouchehrinia, Mohsen Khademi, Michal Bar, Elisabeth Sandberg, and Russell Ouellette declare no conflict of interest.

### PEER REVIEW

The peer review history for this article is available at https://publons.com/publon/10.1002/brb3.2873.

## Supporting information


**S1 TABLE**. Inclusion and exclusion criteria for the ProTEct‐MS study participants
**S2 TABLE**. Definition of diagnostic groups

## Data Availability

The data that support the findings of this study are available from the corresponding author upon reasonable request.
