# Supplementary material for: Demographic and disease‐related factors impact on cerebrospinal fluid neurofilament light chain levels in multiple sclerosis
Source: Brain Behav. 2022 Dec 27;13(1):e2873. doi: 10.1002/brb3.2873 (PMC9847611; doi:10.1002/brb3.2873)
Supplement: Supplementary file 1 — S1 TABLE. Inclusion and exclusion criteria for the ProTEct‐MS study participants S2 TABLE. Definition of diagnostic groups [file BRB3-13-e2873-s001.docx]

**Supplementary methods**

**S1 TABLE. Inclusion and exclusion criteria for the ProTEct-MS study participants**

| Inclusion criteria | 1. The subject has given written informed consent to participate in the study  2. Current diagnosis of RMS, based on the McDonald 2017 criteria  3. Having received treatment with rituximab, as per local clinical routine for at least 12 months prior to the Screening Visit  4. Having received their last dose of rituximab not more than 8 weeks and not less than 4 weeks before Randomization (Study Day 1)  5. Having B-cell count < Lower limit of detection (LLOD) (<0.01*10^-9^ CD19^+^ cells/L)  6. Having Expanded Disability Status Scale (EDSS) 2.5 – 5.5 inclusive at Screening  7. Present clinical worsening in one or more neurological domains as assessed by EDSS, ambulatory function as assessed by 6MWT or T25FW, cognitive functioning as assessed by SDMT or increased need of walking aids or pharmacological/procedures for bowel and bladder functions over the last year  8. Brain Magnetic resonance imaging (MRI), lesion burden with >9 T2 cerebral lesions (assessed within the last 24 months)  9. Stable clinical presentation of MS for 30 days prior to Screening e.g. no relapse, no acute neurological exacerbation  10. Age range from 18 to 55 years (both inclusive)  11. Body weight between 40 – 100 kg (both ranges are inclusive)  12. No disease modifying therapies (DMTs) other than rituximab, within 12 months of Screening  13. No contraindication to Biomarker assessments: brain Magnetic resonance imaging (MRI), blood/serum collection and cerebrospinal fluid (CSF) collection  14. Agreeing to undergo two lumbar punctures  15. Be willing and able to follow all study procedures and assessments according to the study protocol  16. Female patients of childbearing potential (FPCBP) or procreative male patients (PMP), willing to use highly effective contraceptive methods throughout the study duration and at least until 5 months after the last study treatment. The investigators must inform the participant about the risks not to use an effective method of birth control during the course of the study and they should discuss with the participant the most appropriate method.   - FPCBP means female patients who are neither menopausal (for a minimum of at least 2 years), nor underwent irreversible surgical procedures leading to permanent infertility like bilateral tubal occlusion or hysterectomy; - PMP means male patients who have a female partner of childbearing potential and who are not vasectomized (vasectomy to have been a minimum of 10 weeks ago) or underwent other surgical procedures leading to permanent infertility; - Partners of childbearing potential (PCBP) means female partners of a PMP who are neither menopausal (for a minimum of at least 2 years), nor underwent irreversible surgical procedures leading to permanent infertility like tubal ligation or hysterectomy; - FPCBP and PMP, as well as non-pregnant PCBP, must use an effective method of birth control, as described below, (additional local requirements may apply) throughout the study and for at least 5 months after the last dose of study treatment:   - For FPCBP or PCBP, highly effective methods of birth control refer to those which result in a low failure rate (i.e. less than 1% per year), when used consistently and correctly, such as combined hormonal contraception associated with inhibition of ovulation (oral, intravaginal, transdermal), progestogen-only hormonal contraception associated with inhibition of ovulation (oral, injectable, implantable), some intra uterine devices (IUDs), intrauterine hormone-releasing system (IUS), true sexual abstinence (when this is in line with the preferred and usual lifestyle of the participant), or male partner sterilization (vasectomy to have been a minimum of 10 weeks ago);   - For PMP, the barrier method of contraception, condom is considered as acceptable |
| --- | --- |
| Exclusion criteria | 1. Current diagnosis of primary progressive MS (PPMS)  2. Any disease other than MS (e.g. myelitis and /or bilateral optic neuritis) that could better explain the patient’s signs and symptoms  3. Usage of any of the following medications prior to the Screening visit:   1. Any usage of interferon beta, glatiramer acetate, IV immunoglobulin (IVIG), dimethyl fumarate or teriflunomide within 12 months prior to Screening, 2. Any history of exposure to mitoxantrone, cladribine, alemtuzumab, cyclophosphamide, systemic cytotoxic therapy, total lymphoid irradiation, and/or bone marrow transplantation at any time, 3. Any usage of natalizumab within 24 months prior to Screening, 4. Any usage of highly potent immune modulating therapy, such as: ocrelizumab, ofatumumab, fingolimod, siponimod, ozanimod or anticytokine therapy, plasmapheresis or azathioprine within 12 months prior to Screening, 5. Any usage of any experimental treatment if not washed out for ≥ 5 halflives or ≥ 12 months (whichever is longer), except rituximab which is allowed before the study.   4. CTCAE Grade 2 or greater lymphopenia  5. Any major medical or psychiatric disorder that would affect the capacity of the patient to fulfill the requirements of the study, including:   1. Diagnosis or history of schizophrenia 2. Current diagnosis of moderate to severe bipolar disorder, major depressive disorder, major depressive episode, history of suicide attempt, or current suicidal ideation 3. Current or past (within the last 2 years) alcohol or drug abuse   6. History or presence of serious or acute heart disease such as uncontrolled cardiac dysrhythmia or arrhythmia, uncontrolled angina pectoris, cardiomyopathy, or uncontrolled congestive heart failure (NYHA class 3 or 4)  7. Known inability to undergo an MRI scan  8. Contraindications to the use of glucose 5% infusion  9. Inability to follow study instructions, or complete study assessments, as defined by the protocol  10. Any history of cancer with the exceptions of basal cell carcinoma and/or carcinoma in situ of the cervix, and only if successfully treated by complete surgical resection, with documented clean margins and any medically unstable condition as determined by the investigator  11. Legal incapacity or limited legal capacity  12. Pregnant or breastfeeding women  13. History of, or positive serology for viral hepatitis B not explained by vaccination  14. History of, or positive serology for viral hepatitis C or human immunodeficiency virus (HIV) at any time  15. Abnormal liver function tests: AST or ALT > 2 times upper limit of normal range (ULN), or conjugated bilirubin > 2 times ULN, or AP or GGT > 3 times ULN  16. Positive pregnancy test at any time A FPCBP cannot be included in the study if any of the following occurs:   - The urine dipstick pregnancy test indicates a positive result and the pregnancy has not yet been ruled out by the subsequent blood test - No urine dipstick pregnancy test has been performed |

**S2 TABLE. Definition of diagnostic groups**

| Diagnostic groups | Definition |
| --- | --- |
| HCs (n = 89) | Volunteers without specific medical complaints invited only for research purposes. |
| SCs (n = 129) | People reporting neurological symptoms, with following neurological examination and diagnostic tests without relevant alterations.  Symptoms reported were: cognitive difficulties (n = 3), dizziness (n = 5), dysphonia (n = 1), HIV (n = 1), headache (n = 20), headache and sensory disturbance (n = 1), hearing loss (n = 1), idiopathic Bell's facial palsy (n = 2), left leg monoparesis (n = 1), muscular fatigue and back pain (n = 1), myalgia and back pain (n = 1), pain syndrome and headache (n = 3), polymorphic complaints (n = 1), sensory disturbance (n = 63), sensory disturbance and pain (n = 1), sensory disturbance and sensorineural hearing loss (n = 1), sensory disturbance and tinnitus (n = 2), sensory disturbance and hypothyroidism (n = 1), tinnitus (n = 2), trigeminal deficit (n = 1), unspecific neurological symptoms and rheumatological disorders (n = 5), unspecific neurological symptoms with nonspecific white matter changes (n = 3), vertebrogenic syndromes (n = 5), vertebrogenic syndrome and headache (n = 1), visual disturbances (n = 3). |
| NINDCs (n = 122) | This group included the following diagnoses: alcohol-related spastic paraparesis (n = 1), backbone sarcoma (n = 1), bilateral papillary oedema (n = 1), CNS expansions (n = 7), CSF flow abnormalities (n = 7), chronic status after myelitis (n = 1), cortical dysplasia and emotional disorder (n = 1), degenerative white matter changes and cognitive difficulties (n = 1), degenerative white matter changes and cognitive difficulties, fatigue, and pain (n = 1), dementia and Parkinson syndromes (n = 2), familiar hypermetabolism (n = 1), hereditary encephalopathy (n = 1), heroin-induced myelitis (n = 1), mechanical myelitis (n = 1), neurodegenerative diseases (n = 3), neuromuscular bladder dysfunction (n = 1), non-inflammatory PNS involvement (n = 15), nonspecific white matter changes (n = 5), post-concussion syndrome (n = 3), psychiatric disorders (n = 45), sensory disturbance and B12 deficiency (n = 2), sleep disorders (n = 2), spinal cord injury and chemical meningitis (n = 1), spinal injury (n = 1), spinal stenosis and white matter changes (n = 1), spinocerebellar ataxia (n = 1), tethered cord syndrome (n = 1), toxic encephalopathy (n = 1), unconsciousness (n = 1), vascular diseases (n = 10), white matter changes and headache (n = 1), white matter changes, pain, and sensory disturbance (n = 1). |
| MS (n = 415) | This group included people with RRMS (n = 357) SPMS (n = 44), and PPMS (n = 14) |

Abbreviations: n: number; HCs: healthy controls; SCs: symptomatic controls; NINDCs: non-inflammatory disease controls; MS: multiple sclerosis; CSF: cerebrospinal fluid; RRMS: relapsing-remitting multiple sclerosis; SPMS: secondary progressive multiple sclerosis; PPMS: primary progressive multiple sclerosis.
